# Personalized prognosis stratification of newly diagnosed glioblastoma applying a statistical decision tree model

**DOI:** 10.1007/s11060-024-04683-6

**Published:** 2024-04-19

**Authors:** Katharina Conrad, Ronja Löber-Handwerker, Mohammad Hazaymeh, Veit Rohde, Vesna Malinova

**Affiliations:** Department of Neurosurgery, University Medical Center Göttingen, Robert-Koch Straße 40, 37075 Göttingen, Germany

**Keywords:** Glioblastoma, Prognostic factors, Survival analysis

## Abstract

**Purpose:**

Glioblastoma (GBM) is the most frequent glioma in adults with a high treatment resistance resulting into limited survival. The individual prognosis varies depending on individual prognostic factors, that must be considered while counseling patients with newly diagnosed GBM. The aim of this study was to elaborate a risk stratification algorithm based on reliable prognostic factors to facilitate a personalized prognosis estimation early on after diagnosis.

**Methods:**

A consecutive patient cohort with confirmed GBM treated between 2010 and 2021 was retrospectively analyzed. Clinical, radiological, and molecular parameters were assessed and included in the analysis. Overall survival (OS) was the primary outcome parameter. After identifying the strongest prognostic factors, a risk stratification algorithm was elaborated with estimated odds of survival.

**Results:**

A total of 462 GBM patients were analyzed. The strongest prognostic factors were Charlson Comorbidity Index (CCI), extent of tumor resection, and adjuvant treatment. Patients with CCI ≤ 1 receiving tumor resection had the highest survival odds (88% for 10 months). On the contrary, patients with CCI > 3 receiving no adjuvant treatment had the lowest survival odds (0% for 10 months). The 10-months survival rate in patients with CCI > 3 receiving adjuvant treatment was 56% for patients younger than 70 years and 22% for patients older than 70 years.

**Conclusion:**

A risk stratification algorithm based on significant prognostic factors allowed a personalized early prognosis estimation at the time of GBM diagnosis, that can contribute to a more personalized patient counseling.

## Introduction

Glioblastoma (GBM) is the most aggressive glioma recurring on a regular basis despite extensive tumor treatment and usually resulting into a limited overall survival (OS) [1]. Although GBM is a clearly defined entity on a histological and molecular level, the affected patients often exhibit a heterogenous manifestation regarding clinical and radiological characteristics, which may impact the survival odds on an individual basis [2]. An individual prognosis estimation at the time of diagnosis is of great relevance not only for treatment decision-making but also for counseling patients and their relatives. Several prognostic factors of GBM have been identified in the past years. Younger age, a better clinical status at diagnosis, tumor location outside of eloquent regions, gross total resection (GTR) of the tumor, and promotor methylation of the O-6-methylguanine-DNA methyltransferase (MGMT) count to the most relevant prognostic factors in GBM patients [3–5]. The current World Health Organization (WHO)-classification of 2021 represents a conceptual progress in the diagnosis of GBM based on molecular parameters [5]. Despite a few previously reported attempts to establish a staging system for GBM based on imaging parameters to facilitate an estimation of achievable extent of resection [6, 7], no prognostication system has been established yet weighting up prognostic factors for a more personalized prognosis estimation in clinical practice. In this study, we aimed to develop a prognostication tool for GBM patients based on objective clinical, radiological, and molecular factors with high prognostic value, that are available early on after the diagnosis of GBM. This tool should facilitate an approximation of the individual probability of survival in patients with newly diagnosed GBM considering the individual risk factors constellation of the patient.

## Methods

### Study population and study design

This is a retrospective observational study. A consecutive patient population with newly diagnosed GBM in the time from 2010 to 2021 was analyzed. Only patients with confirmed diagnosis of GBM, who were treated at our center beginning from the day of tumor diagnosis and followed up until the day of death, were included in the study. Considering the study period between 2010 and 2021 the GBM diagnosis was made according to the valid classification system for brain tumors at that time. For accordance reasons with the new classification 2021, tumor with IDH mutation were excluded from the analysis.

### Tumor treatment

The decision to perform a tumor biopsy or tumor resection was made depending on the tumor manifestation on imaging. Tumors primarily involving key brain structures like the corpus callosum, the basal ganglia, the brain stem, or other eloquent regions as well as tumors with multifocal/multicentric tumor manifestation received a tumor biopsy. Furthermore, a distinction between subtotal resection (STR) and GTR was made. GTR was assigned as soon as the extent of resection was ≥ 95% of the tumor on contrast-enhanced T1-weighted sequence. The extent of resection was assessed by magnetic resonance imaging (MRI) 72 h after surgery. After diagnosis confirmation the adjuvant treatment was interdisciplinary discussed in the institutional tumor board for tumors of the central nervous system. The adjuvant treatment consisted in most cases of radio-chemotherapy according to the Stupp protocol. A small proportion of patients received radiotherapy only or were included in clinical trials, that were ongoing at the time of GBM diagnosis. These studies included the CeTeG trial, the GLARIUS trial, the CENTRIC trial, the NOA-08 trial and the procabazine, lomustine, and vincristine (PCV) regimen [8–11].

### Considered prognostic factors and outcome parameters

The primary outcome parameter was OS defined as the time from the date of GBM diagnosis to the date of death. The following clinical parameters were evaluated: age at diagnosis, sex, clinical symptoms at the initial manifestation as well as at the time of recurrence. Epileptic seizures, focal neurological deficits, cognitive deficits, and the occurrence of headache were considered. Focal neurological deficits included aphasia, paresis, and visual disturbances. Cognitive deficits were defined as memory and concentration disorders as well as behavioral changes. In addition, the Charlson Comorbidity Index (CCI) was calculated to consider present comorbidities at diagnosis [12]. The following comorbidities are considered in the CCI: 1 point was assigned to myocardial infarction, cardiac insufficiency, peripheral artery occlusive disease, cerebrovascular disease, dementia, chronic pulmonary disease, connective tissue disease, peptic ulcer disease, mild hepatic disease, and diabetes mellitus, respectively; 2 points were assigned for hemiplegia, moderate to severe renal disease requiring dialysis, diabetes mellitus with further organ dysfunctions, non-metastatic tumor, leukemia, and lymphoma, respectively; 3 points were assigned for moderate to severe hepatic disease i.e., liver cirrhosis, and 6 points were assigned for a metastatic solid tumor or an acquired immunodeficiency syndrome. Additional points were assigned according to the following age groups: 1 point for an age of 50–59 years, 2 points for an age of 60–69 years, 3 points for an age of 70–79 years, 4 points for an age of 80–89 years, and 5 points for an age of 90–99 years [12]. The recurrence date was defined as the date of the MRI documenting a tumor recurrence. The preoperative Karnofsky performance status (KPS) and that at recurrence were considered as well. An evaluation of the following radiological tumor characteristics was performed: tumor size, tumor extension and tumor localization were analyzed based on the initial MRI using the contrast-enhanced T1-weighted sequence. The tumor volume was calculated applying the ABC (A = length, B = width, C = height) / 2 formula, considering the contrast-enhanced tumor. Based on their manifestation on imaging, the tumors were divided into three groups: singular tumors (one coherent tumor mass located within one or more brain lobes), multifocal tumors (multiple tumor lesions with a distance of < 1 cm to each other and a visible connection between lesions on T2-sequence) and multicentric tumors (multiple distant tumor lesions without a visible connection between lesions). Furthermore, a ventricular contact as well as an involvement of the peri-trigonal area were documented. In case of tumor recurrence, a differentiation was made concerning a local or a distant recurrence. Data about molecular markers were extracted from the neuropathological findings of the patients. The presence of p53 and IDH mutations or the methylation of the MGMT promotor, and the proliferation marker Ki67 were considered. Since molecular markers were routinely performed at our center starting in 2016, data on molecular markers were available only in the patient population from 2016 to 2021.

### Statistical analysis

The statistical analyses were performed by means of the GraphPad Prism software (Version 9, GraphPad Software, San Diego, CA, USA). Furthermore, IBM SPSS statistics (Version 28.0) was used. For the presentation of baseline data descriptive statistics was done. Continuous variables were depicted as mean ± standard deviation (SD), categorical variables as frequency or percentages. Fisher’s exact test was performed to calculate odds ratios (OR), sensitivity and specificity. A systematic analysis of the patient cohort was performed using single Cox regression analyses. Variables were related to survival time and analyzed for their effect. The significance level was set at a p-value ≤ 0.05. The Hazard ratio, which indicates the probability that an event will occur in each time, was determined to examine the effect of each significant parameter on survival. A Hazard ratio < 1 was associated with longer survival, whereas a Hazard ratio > 1 indicated a shorter survival. The 95% confidence interval (CI) was also determined to examine whether the variables indeed generated a change in survival. The Benjamini-Yekutieli correction was applied to avoid alpha error accumulation. Then a multivariate Cox model was constructed using the significant factors from the univariate analyses. The classification tree was constructed, and parameters known at the time of diagnosis were incorporated into the statistical model. Patients with missing data were censored from statistical analysis. The classification tree was created using the SPSS statistics software (IBM SPSS statistics Version 28.0). The preoperatively recorded parameters were set in relation to the survival time in months. The classification tree procedure is used to create a tree-based classification model. The cases are classified in groups according to the dependent variable (target variable) and are predicted based on the values of independent variables (influencing variables). The tree was created using the CHAID (Chi-squared Automatic Interaction Detection) method, which facilitates an automatic detection of correlations using chi-squared tests. In each step, the CHAID method determines the independent variable that shows the strongest correlation with the dependent variable. Furthermore, the cross-validation procedure was used, which then builds a single final tree model. The cross-validated risk estimate for the final tree is calculated as the average of the risks for all trees. The mean value and the standard deviation as well as median values and 95%CI for the survival time were calculated in the tree in a standardized manner.

## Results

### Patient characteristics

A total of 462 consecutive patients with confirmed diagnosis of GBM and treated at our center in the time between 2010 and 2021 were enrolled in the study. The mean age at GBM diagnosis was 65 ± 13 years, 58% (269/462) of the patients were male. The mean KPS in the patient cohort was 77 ± 14% and the mean CCI was 3 ± 2. The most frequently found symptoms at manifestation were focal neurological deficits (64%), followed by cognitive deficits (43%), cephalgia in 22%, and epileptic seizure in 18% of the patients. The achieved extent of tumor resection was as followed: GTR in 57% (261/462), STR in 25% (116/462), and biopsy in 18% (85/462). Adjuvant treatment was performed in 91% (408/462), 8% (34/462) rejected to receive adjuvant treatment, and 1% (4/462) of patients died before starting an adjuvant treatment.

### Tumor characteristics

In 74% (344/462) of patients GBM manifested as a singular tumor and 118 patients had GBM with multifocal/multicentric presentation (39 multicentric, 79 multifocal) on initial MRI. A tumor location in eloquent brain regions was found in 31% (144/462) of cases, in 67% (308/462) the tumor had contact to the ventricular system and of 33% (101/308) of these patients had a peri-trigonal location. Table 1 gives an overview of the baseline characteristics in the study population. A MGMT-promotor methylation had 43% of tumors and all included tumors were IDH wildtype. A mutation of p53 was present in 65% of tumors. The mean Ki67-proliferation index of tumors was 12 ± 7%.

**Table 1:** Baseline characteristics

Variables	All
**Number of patients (%)**	462 (100%)
**Mean age ± SD in years**	64.6 ± 13
**Sex**	
Male (%)	269 (58.2%)
Female (%)	193 (41.8%)
**Mean KPS preOP ± SD in %**	77.0 ± 14%
**Focal neurological deficit at diagnosis (%)**	295 (64%)
**Epileptic seizures (%)**	81 (18%)
**Cognitive deficits at diagnosis (%)**	193 (42%)
**Charlson comorbidity index**	
Mean ± SD	2.7 ± 1.9
≤ 1 point (%)	119 6%)
1–3 points (%)	203 (44%)
> 3 points (%)	140 (30%)
**Molecular markers**	
MGMT methylation	88/205 (43%)
p53	78/223 (35%)
Ki67	11.8 ± 8%
**Tumor manifestation**	
Singular tumor	74% (344/462)
Multilocular tumor	26% (118/462)
**Extent of resection**	
Gross total resection	57% (261/462)
Subtotal resection	25% (116/462)
Biopsy	18% (85/462)
**Adjuvant treatment**	
Stupp protocol	72% (332/462)
CeTeG protocol	6% (26/462)
Radiotherapy only	6% (29/462)
Other protocol	4% (21/462)
No treatment	12% (54/462)

### Predictors of survival– a decision tree for an early prognosis estimation

**Fig. 1:**
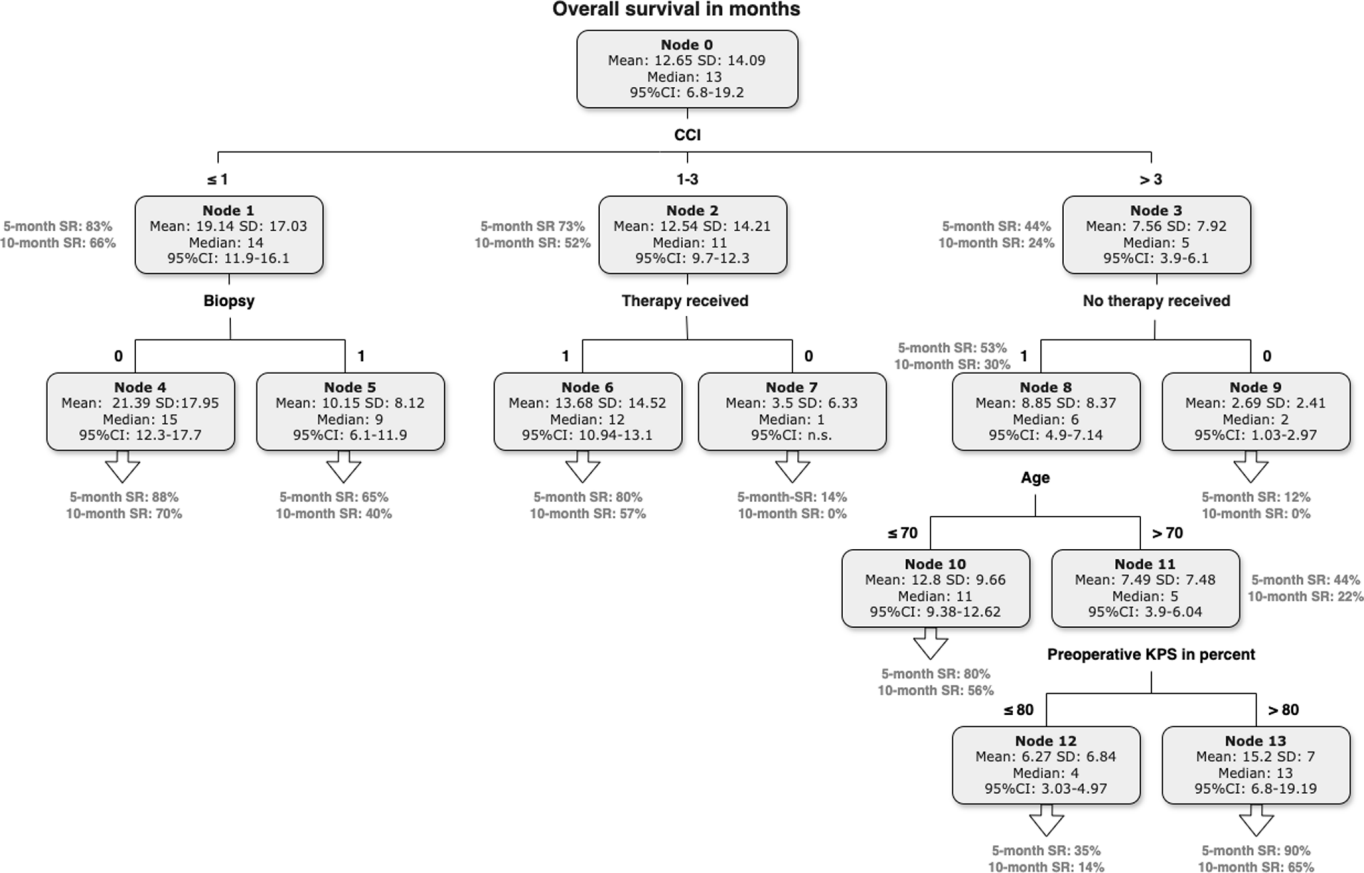
Decision tree stratifying the probability of survival dependent on the individually present prognostic factors starting with the prognostic factor with the highest discriminatory power (CCI) and allowing a stepwise survival rate (SR) stratification including further prognostic factors (extent of tumor resection and adjuvant treatment)

Data concerning OS were available in 87% (404/462) of all patients. At the time of data acquisition 8% (37/462) of patients were alive. In 5% (21/462) of patients, no data were available concerning OS. The median OS in the study population was 9 months (95%CI 7–10), range 1-124 months. While KPS at manifestation as well as at recurrence was a positive predictor of longer OS (Table 2), higher age, the presence of significant comorbidities (CCI > 3), and focal neurologic deficits at presentation were identified as negative predictors of OS (Table 3). A summary of the treatment for each group of CCI is given in Table 4. In the multivariate model the presence of comorbidities according to CCI, focal neurological deficits, the primary location of the tumor within corpus callosum, and a primary palliative treatment approach remained independent predictors of OS (Table 5). The statistically elaborated decision tree including the strongest survival predictors is depicted in Fig. 1. The CCI was the strongest survival predictor in our study cohort, hence, represented the main subdivision criterion in the decision tree. Patients without or with only few comorbidities (CCC ≤ 1) had a significantly higher mean OS (19 ± 16 months), than those with moderate number of comorbidities (CCI 1–3) with a mean OS of 12 ± 14 months, and patients with significant comorbidities (CCC > 3) with a mean OS of 7 ± 8 months (*p* < 0.0001). The next subdivision criterion was the extent of resection. Patients who received STR or GTR had significantly higher OS rates compared to those who underwent only biopsy (21 ± 17 vs. 10 ± 8 months). The survival rate in the patient group with STR or GTR was 88% after five months, and 70% after 10 months. On the other hand, the survival rate of patients with biopsy only was 65% after five months and 40% after ten months. The next subdivision criterion in the patient group with moderate comorbidities was whether the patients received adjuvant treatment or not. While the mean OS in the patient group receiving adjuvant treatment was 14 ± 15 months, the patients without adjuvant treatment had a mean OS of 4 ± 6 months. In the patient group (124/404 patients) with significant comorbidities (CCI > 3), age and the preoperative clinical status had allowed a further stratification of OS in each patient group. The OS in the patient group with adjuvant treatment and age older than 70 years, the clinical status at presentation was the decisive criterion with a survival rate of 90% after five months and 65% after ten months for patients with KPS > 80% compared to 35% after five months and 14% after ten months for patients with KPS ≤ 80%.

**Table 2:** Positive predictors of OS

Parameter	p-value	Hazard ratio	95%CI
KPS	< 0.001	0.98	1.02–1.03
KPS recurrence	< 0.001	0.98	0.97–0.99
Number of performed operations	< 0.001	0.46	0.37–0.58
GTR recurrence	< 0.001	0.53	0.39–0.72
PFS	< 0.001	0.95	0.94–0.97
Stupp protocol	< 0.001	0.49	0.39–0.61
Singular tumors	0.01	0.78	0.64–0.95

**Table 3:** Negative predictors of OS

Parameter	p-value	Hazard ratio	95%CI
Age	< 0.001	1.03	1.02–1.03
CCI	< 0.001	1.2	1.12–1.27
Focal neurologic deficits	< 0.001	1.52	1.12–1.87
Biopsy	< 0.001	1.67	1.3–2.14
Palliative therapy	< 0.001	5.22	3.56–7.67
Multifocal GBM	< 0.001	1.77	1.31–2.39
Involvement of corpus callosum	< 0.001	1.72	1.34–2.21
Ventricular contact	< 0.001	1.46	1.17–1.81

**Table 4:** Summary of the treatment for each group of CCI

Variables	CCI 0 or 1	CCI 2 or 3	CCI > 3	p-value
Received tumor treatment % (n)				0.02
- Yes	94% (112/119)	90% (183/203)	81% (113/140)	
- No	6% (7/119)	10% (20/203)	19% (27/140)	
Received Stupp protocol % (n)				0.02
- Yes	77% (92/119)	80% (163/203)	55% (77/140)	
- No	23% (27/119)	20% (40/203)	45% (63/140)	
Received CeTeG protocol % (n)				0.001
- Yes	13% (15/119)	4% (9/203)	1% (2/140)	
- No	87% (104/119)	96% (194/203)	99% (138/140)	
Received GTR % (n)				0.154
- Yes	50% (59/119)	57% (116/203)	61% (86/140)	
- No	50% (60/119)	43% (87/203)	39% (54/140)	
Received STR % (n)				0.08
- Yes	33% (39/119)	22% (44/203)	24% (33/140)	
- No	67% (80/119)	78% (159/203)	76% (107/140)	
Received biopsy % (n)				0.338
- Yes	18% (21/119)	21% (43/203)	15% (21/140)	
- No	82% (98/119)	79% (160/203)	85% (119/140)	

GTR = gross total resection, STR = subtotal resection, CCI = Charlson Comorbidity Index

**Table 5:** Multivariate model

Parameter	p-value	Hazard ratio
Focal neurologic deficits	0.001	1.52
CCI	0.017	1.11
Palliative treatment approach	0.007	2.10
Primary corpus callosum	0.042	1.65

## Discussion

In this observational study, a large consecutive cohort of GBM patients was analyzed and a risk stratification algorithm based on the strongest prognostic factors was elaborated to facilitate a more personalized survival odds estimation at the time of diagnosis confirmation. Clinical, radiological, and molecular prognostic factors were included into the decision tree analysis. The presence of comorbidities assessed with the CCI was the strongest prognostic factor stratifying the patient cohort in three survival groups. Patients with no or mild comorbidities (CCI ≤ 1) had the highest odds of longer survival, followed by the patients with moderate comorbidities (CCI 2–3), and the patients with severe comorbidities (CCI > 3), who had the lowest odds of longer survival. Although other prognostic factors such as age and clinical status at presentation have been more often used in clinical practice, it is not surprising that comorbidities also play an important role for prognosis estimation in GBM patients. Frailty and comorbidity burden have gained attention in recent years with increasing number of publications outlining the importance of these factors for GBM patients [13–15]. The CCI was originally developed for the estimation of mortality in cancer patients [12]. According to the findings of our study, comorbidities seem to play a crucial role for the prediction of survival in GBM patients as well. Regarding the distribution of comorbidities in our study cohort with no or mild comorbidity in 23% of cases, moderate comorbidities in 44% of cases, and severe comorbidities in 33% of patients shows a wide distribution of comorbidities in GBM patients. Additionally, the average manifestation age of GBM patients is comparable with the average manifestation age of other cancer types in adults. Therefore, CCI seems to be a suitable parameter to be considered while estimating the survival odds in patients with newly diagnosed GBM. Aside from CCI, the extent of tumor resection and the conducted adjuvant tumor treatment were the most decisive factors in our study cohort. Patients undergoing only tumor biopsy instead of tumor resection died significantly earlier (11.2 months), even if they were younger. This highlights the relevance of extent of reasonably achievable tumor resection. In the classification tree, no categorization was made into STR and GTR, but only into biopsied and no biopsied patients. The effect of biopsy in our study cohort was obviously of greater importance in terms of OS than the difference of STR to GTR. The findings of our study are indicating a life-prolonging effect of STR compared to performing only a tumor biopsy. Since GTR is the gold standard for achieving a survival benefit for the patients, this finding is controversial in comparison to the common literature [16–18]. The consideration of age and initial KPS allowed a further stratification of survival odds in subgroups of patients but showed a lower discrimination power than CCI, which may be the consequence of a previously reported relationship between age, CCI, KPS and treatment capacity of the patients [19–22]. There is a trend toward performing only tumor biopsy in older patients in clinical practice. Additionally, patients older than 70 years are often not considered able to receive full adjuvant tumor treatment. Currently, GBM patients older than 70 years are usually treated with hypofractionated radiotherapy with or without additional temozolomide [23]. In our study, the preoperative KPS played a crucial role for further survival stratification in patients older than 70 years, that should be considered in a personalized treatment counseling of older patients in clinical practice. A KPS greater than 80% was associated with nine months longer OS, even when patients had an age greater than 70 years. This suggests that tumor therapy can significantly improve the prognosis of patients even at older ages given they exhibit a good functional status at presentation [21]. Patients with multiple comorbidities are mostly older and have a lower KPS. Thus, these parameters could be cofounders. Comorbidities are more often found in older patients, which may also contribute to a reduced functional status resulting into a reduced KPS. Hence, comorbidities and the functional status may prevent the patients from being able to receive a more aggressive tumor treatment, which in turn can result into a shorter OS [1, 21, 22].

The first attempt of addressing the need for a prognosis stratification tool to be used in clinical practice was made 1993 by the Radiation Therapy Oncology Group (RTOG) applying recursive partitioning analysis (RPA) based on clinical parameters such as age, KPS, and the extent of tumor resection [24]. The first RPA classification divided malignant gliomas (including not only patients with GBM but also patients with anaplastic astrocytoma) into six classes with distinctive survival outcomes dependent on the above-mentioned parameters. The patients included in this analysis were treated in a period before the temozolomide became a treatment of choice for patients with a newly diagnosed GBM [3]. The European Organization for Research and Treatment of Cancer (EORTC) Group modified the original RTOG RPA classification demonstrating that the addition of temozolomide to radiotherapy provided a survival benefit [25]. The tumor resection boundaries also have changed over time leading to fluorescence-guided glioma resection becoming a standard procedure [16–18]. Pichlmeier et al. have considered this aspect in RPA analysis and demonstrated a significantly longer overall survival of patients with complete tumor resections compared to their counterparts with subtotal resections, which was found to be the case for the RTOG-RPA class IV and V [26]. Furthermore, the prognostic role of molecular markers such as IDH mutations and the MGMT promotor methylation has been increasingly acknowledged over the past years. Finally, this led to the integration of established molecular markers in the last World Health Organization (WHO) classification of gliomas in 2021 [5]. For this reason, modifications of the original RTOG RPA classification were done in the last years. Wee et al. added the IDH mutation and the MGMT promotor methylation to the RTOG RPA classification, where the MGMT status was indeed the strongest node in the classification tree of their study followed by the IDH mutation [27]. According to the new WHO-classification for glioma, malignant gliomas with an IDH mutation are not considered GBMs anymore, hence, the use of the IDH mutation for stratification of the survival odds in GBM patients does not meet the current criteria for GBM diagnosis. On the other hand, Bell et al. chose a different approach and performed measurements of 22 proteins’ expression associated with GBM pathogenesis and investigated whether they can increase the discriminatory power of the existing RTOG RPA classification [28]. The MGMT protein expression within the tumor was found to exhibit a stronger effect on survival compared to the MGMG promotor methylation. The Ki67, cMet, and the MGMT protein have been found to have the highest impact on survival in the study of Bell et al. [28]. The approach of our study was to elaborate a decision-tree model based on clinical and radiological parameters available at the time of diagnosis but also considering the resectability of the tumor and the ability of the patients to receive adjuvant tumor treatment. While previous studies have also used age and KPS as clinical parameters, CCI was not only a new additional parameter which was applied in our study but showed also the strongest discriminatory power concerning the survival odds of patients with newly diagnosed GBM according to the new WHO-classification. The molecular markers showed a weaker role in the decision tree model compared to the other parameters such as CCI, KPS, and the extent of resection. However, molecular parameters were available only in the half of the study population (2016 to 2021 molecular parameters were evaluated on a regular basis in our hospital), which might have influenced the results of our study. Woo et al. evaluated several existing survival stratifications tools and found that none of the models resulted in an ideal agreement between predicted and observed 12-months survival odds, but the RTOG nomogram model performed best in this comparative study [29]. The statistically elaborated decision tree method in our study was able to balance the relationship between these prognostic factors allowing a reliable stratification of survival odds on an individual basis. Therefore, the individual interplay of prognostic factors is more important than a decision based only on one parameter like age.

## Limitations of the study

The main limitation of the study is the retrospective study design with incomplete data concerning the molecular markers. However, a large cohort of GBM patients was analyzed followed up from the diagnosis confirmation to the date of death allowing the calculation of OS in 95% of included patients. Since the decision tree model was build based on the analyzed patients of our study cohort, who was treated in our center from the GBM diagnosis to the day of death, not including patients treated at other center, we the question remains unanswered, whether the decision tree model will provide the same results in an external patient cohort. The validity of the decision tree would need an external validation to establish it as a tool to be used in clinical practice.

## Conclusion

An elaborated decision tree algorithm reflecting the individual constellation of prognostic factors allowed a reliable prognosis estimation in patients with newly diagnosed GBM. The CCI was the strongest prognostic factor in our study stratifying the patient cohort into three groups with different survival probability dependent on the presence and severity of comorbidities, tumor resectability, and the ability to receive tumor treatment. A prospective validation of the decision tree method presented in our study is needed including multiple centers to confirm its usefulness as a prognostic tool in clinical practice.

## Data Availability

All generated and analyses data are presented in the manuscript.

## Abbreviations

CI: confidence interval
GBM: glioblastoma
GTR: gross total resection
IDH: isocitrate dehydrogenase
KPS: Karnofsky performance score
MGMT: O-6-methylguanine-DNA methyltransferase
MRI: magnetic resonance imaging
OR: odds ratio
OS: overall survival
PFS: progression free survival
SD: standard deviation
STR: subtotal resection
WHO: World Health Organization
